# Immersive medical training: a comprehensive longitudinal study of extended reality in emergency scenarios for large student groups

**DOI:** 10.1186/s12909-024-05957-3

**Published:** 2024-09-09

**Authors:** Jonas Einloft, Hendrik L Meyer, Simon Bedenbender, Muriel L Morgenschweis, Andre Ganser, Philipp Russ, Martin C Hirsch, Ivica Grgic

**Affiliations:** 1 Department of Internal Medicine and Nephrology, University Hospital Giessen and Marburg, Philipps University Marburg, Marburg, Germany; 2 Institute for Artificial Intelligence in Medicine, University Hospital Giessen and Marburg, Philipps University Marburg, Marburg, Germany

**Keywords:** Virtual reality (VR), Medical education, Emergency training scenarios, Technology acceptance model, Immersive technologies, Future of teaching, Extended reality (XR)

## Abstract

Young healthcare professionals and medical graduates often fall short in the practical experience necessary for handling medical emergencies. This can not only lead to strained feelings of inadequacy and insecurity among future physicians and less experienced healthcare providers in general, but also to detrimental outcomes for patients as emergency medicine demands rapid decision-making with low tolerance for errors. New didactic modalities and approaches may be needed to effectively address this shortcoming. Immersive technologies are powerful novel educational tools with untapped potential in medical training, and may be particularly suitable for simulation trainings in the high-stakes field of emergency medicine.

Herein, we systematically explored the educational potential of extended reality (XR) technology, particularly virtual reality (VR), in the management of patients presenting as medical emergencies, combining the use of the STEP-VR application with an untethered hardware setup.

Importantly, we aimed at studying multiple, large cohorts of senior medical students involving a total of 529 participants and collecting data over a period of two years. We assessed students’ acceptance of the training through a modified questionnaire measuring device handling, content complexity, degree of immersion, learning success, and seminar design.

Our results show high, sustained acceptance and ease of use across different student cohorts and subgroups, with most students finding XR/VR engaging and beneficial for acquiring emergency medicine skills. Importantly, the prevalence of simulation sickness was minimal. Moreover, no major effect of the head-mounted displays (HMDs) price range was noted with regard to the learning experience. The results underscore the potential of XR/VR capabilities in effectively enhancing medical education, particularly in areas of high-stakes clinical scenarios and emergency care, by providing realistic and reproducible immersive training environments.

In summary, our findings suggest that XR/VR-based training approaches could significantly contribute to preparing future physicians for the complexities of emergency medical care, encouraging the integration of such technologies into medical curricula. However, careful consideration must be given to its suitability for all students and the practical challenges of its implementation, highlighting the need for further research to harness its full potential for medical education.

**Supplementary Information:**

The online version contains supplementary material available at 10.1186/s12909-024-05957-3.

## Background

In the dynamic and often high-stakes field of emergency medicine, the ability to make fast, effective decisions in complex and time-sensitive scenarios is of utmost importance. It demands proficiency in executive functions and task prioritization, skills that are crucial in managing this type of clinical presentation [1].

However, deficits exist in the preparedness of graduates to manage medical emergencies, as evidenced by shortcomings in areas such as clinical reasoning, diagnosis, prescribing, multidisciplinary teamwork, and patient handover [2–5]. This lack of readiness, observed by clinical educators and self-reported by students and graduates alike, underscores the need for more effective educational interventions [4, 6].

To bridge this gap, various training approaches have been employed, ranging from theoretical lessons to skill training and simulation training with patient actors. Yet, the challenges in emergency medicine extend beyond these traditional methods, as they encompass a range of audiovisual stimuli and interpersonal interactions that constitute ‘situational awareness’ [7–9]. This complexity has led to the exploration of extended reality (XR) technologies, which includes virtual reality (VR) and augmented reality (AR), as a potential tool for enhancing the training landscape [10–12]. XR is commonly used as an umbrella term that encompasses all immersive technologies. VR refers to a fully immersive technology that simulates a completely digital environment for the user, typically using headsets. In contrast, AR does not replace the real world but rather adds digital content to the user’s field of vision [13, 14].

XR technology offers a distinct advantage with its immersive capabilities that are currently, for the most part, being provided by head-mounted displays (HMD) and interactive controllers. It facilitates experiential learning and improves contextualization that are beneficial, if not even critical, for acquiring competencies in emergency medicine.

Unlike physical simulators, XR can replicate a higher level of perceived reality, offering repeated practice in rare but critical scenarios such as patients with, or at life-threatening risk of, rapid clinical deterioration. Moreover, XR-based training is considered less resource-intensive than high-fidelity physical simulations, thus making it potentially more accessible and cost-effective [7].

However, the implementation of immersive technology in emergency medicine training at medical schools or teaching hospitals poses challenges [15]. Amongst others, technical and infrastructural constraints, reservations towards technology, aspects of usability and simulation sickness, as well as didactic usefulness and curricular integration must be considered. Hence, the suitability of educational XR for all students – which ultimately ought to be the goal – remains a subject of ongoing research.

To better assess the educational potential of emerging XR technology in medical education, particularly in managing medical emergencies, we aimed to conduct a large-scale study on students’ opinions of XR/VR-simulated emergency patients using untethered state-of-the-art HMD solutions.

The objectives of this study were as follows:

1. To assess the feasibility of XR/VR-based simulation training for managing medical emergencies under our specific conditions.
2. To investigate students’ acceptance of this specific XR/VR-based simulation training, gather their subjective learning feedback, and perform comparative analyses across consecutive cohorts over time.
3. To evaluate the overall seminar design.
4. To assess the occurrence of simulation sickness and determine the suitability of different HMDs for our objectives.

This larger study builds upon and expands the pilot research by Mühling et al., which originally tested the specific learning program with a smaller group of participants [16].

## Methods

### Survey participants

To evaluate the receptiveness of medical students towards VR-based simulation training in the management of medical emergencies, we conducted a study involving 529 senior fifth year medical students at the Philipps University Marburg. As a component of the practical “Internal Medicine” course, the VR-based training was integrated as a curricular seminar targeting students in their 9th or 10th semesters. The study spanned from the winter semester of 2021 to the summer semester of 2023. Each student participated in the study only once.

### VR-HMD and VR application

In this study, we used the Oculus Quest 2 (now Meta Quest 2) HMDs from the winter semester of 2021 through the winter semester of 2022, and its advanced version, the Meta Quest Pro, in the summer semester of 2023. These HMDs are notable for their portability, inside-out tracking, and wireless connectivity. In addition, the Meta Quest Pro offers improved features including increased pixel density and extended field of view [17]. We employed the “STEP-VR” (short for “Simulation-based Training of Emergencies for Physicians using Virtual Reality”) application, originally developed by ThreeDee GmbH in collaboration with the University Würzburg’s Medical School, for simulating an emergency room environment [18]. STEP-VR enables the enactment of various independent emergency scenarios with avatar patients. The software ran on a Predator Helios 3000 laptop (chip: Intel Core i7-1050H, graphics card: NVIDIA GeForce RTX 3080 with 8GB of GDDR6 dedicated VRAM). Wireless streaming to the VR-HMD via WLAN was conducted using Meta Quest Air Link and an Asus AX5400 router.

### Survey design

To evaluate student acceptance of VR-based emergency training, we used a modified version of a previously employed questionnaire [16]. This adapted questionnaire comprised 23 items, assessing VR device handling, content complexity, immersion, subjective learning success, and seminar design (Supplementary Table 1). The responses to the questionnaire items were rated on a 5-point Likert scale, where scores represented 1=strongly disagree, 2 = somewhat disagree, 3=neither agree nor disagree, 4=somewhat agree, and 5=strongly agree. To validate the reliability of our measures, we conducted a reliability analysis using Cronbach’s *α*, employing the *alpha* function from the psych (2.3.9) R package; *α* values greater than 0.6 were considered as acceptable and those above 0.7 were considered as good [19, 20]. Additionally, a simulation sickness questionnaire was used to identify 16 typical simulation sickness symptoms [21]. We also included demographic queries, with three open-ended questions on age, sex, and semester, and a closed-ended question about previous VR experience.

### Study design

**Fig. 1:**
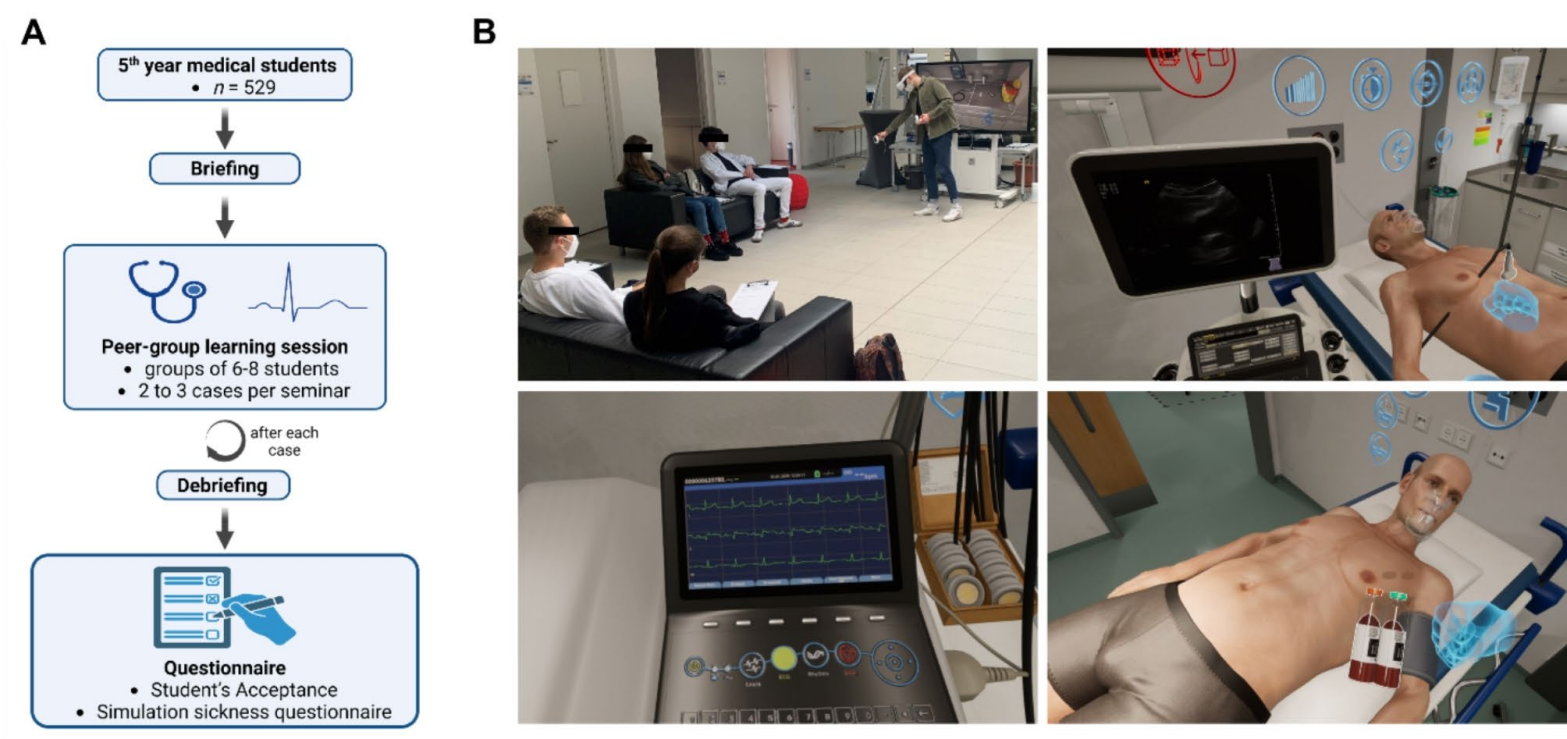
Study design and visual impressions. **A**, Flowchart illustrating the study design and participant progression. **B**, Screenshots depicting the in-simulation experience within STEP-VR

The VR-based simulation training for managing medical emergencies was incorporated into the curricular practical course in internal medicine, specifically targeting fifth-year medical students in their 9th or 10th semester. These sessions were structured as peer-group seminars, facilitated by student tutors who provided both medical insights and technical support. Groups of 6–8 students participated in each session (Fig. 1A).

Each seminar began with a structured briefing and introduction to the HMD and controllers as well as the software. Students then engaged in 2 to 3 of the available cases. In these scenarios, one student interacted with the simulation using the HMD, while the others watched on a screen and collectively discussed potential approaches, diagnostic steps, findings and treatment strategies (Fig. 1B). Each concluded case was followed by a focused debriefing before starting with a new case. At the seminar’s conclusion, participants were given printed surveys to fill out voluntarily.

### Data analysis

The collected survey data, comprising both closed- and open-ended questions, were analyzed using R (version 4.3.0) and RStudio (version 2023.09.1) statistical software. The answers were manually digitized and entered into an Excel spreadsheet, which served as input for the data analysis. Demographic statistics such as age, gender, semester and previous VR experience were calculated using the collected survey data. The Likert analysis was performed using the likert (1.3.5) R package and the resulting data was used to generate Likert plots for each question using the ggplot2 (3.4.3) R package. Differences in self-reported ease of use between the genders were statistically tested by unpaired Mann-Whitney *U* testing using the *pairwise.wilcox.test* function of the stats (4.3.0) package as normality of the data could not be assumed according to Shapiro-Wilk testing. Results were considered statistically significant for a significance level α ≤ 0.05.

## Results

### Demographics

Our study involved a total of 529 medical students, comprising 338 females, 171 males, 3 non-binary, and 17 undisclosed gender identities, with a median age of 24 years (range 21–36 years), as detailed in Table 1. Participation was distributed as follows: 142 students in winter semester 2021, 119 in summer semester 2022, 137 in winter semester 2022, and 131 in summer semester 2023. Of these, 398 utilized the Meta Quest 2 HMD, while 131 used the advanced Meta Quest Pro version. Notably, nearly three-quarters had no prior VR experience. Comprehensive demographic data are available in Table 1.

### Students’ acceptance

**Fig. 2:**
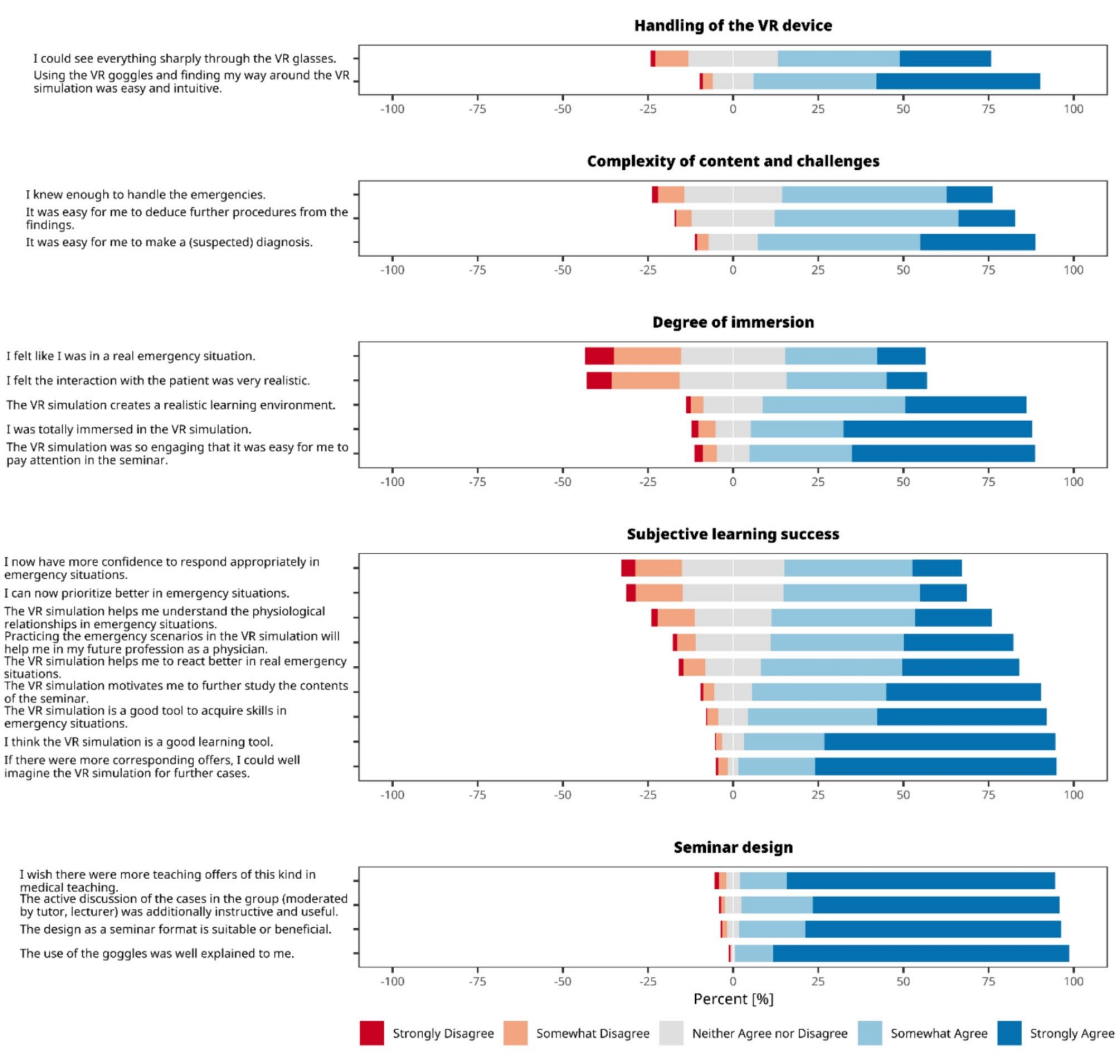
Likert plots (5-point scale) displaying survey results for the five measured criteria: (i) handling of the VR device; (ii) complexity of content and challenges; (iii) degree of immersion; (iv) subjective learning success; and (v) seminar design). Bars represent the percentage distribution of responses for each Likert score

To explore the potential of a XR/VR-based simulation training of medical emergencies, we aimed to evaluate the effectiveness of this novel teaching format by assessing student acceptance, focusing on the following aspects (Fig. 2):

1. Handling of the HMD.

2. Complexity of content and challenges.

3. Degree of immersion.

4. Perceived learning success.

5. Seminar design.

The majority of students (84%) found that using the HMDs and navigating the simulation was both straightforward and intuitive. A small fraction (3.9%) experienced difficulties with the HMD or VR simulation. While a majority (63%) had no issues with clarity of vision through the VR glasses, 11% reported challenges in achieving sharp vision.

Evaluating student responses concerning the content complexity of the VR simulation revealed it was generally well-matched to their skill level. 62% of students felt their existing knowledge was adequate for coping with the emergency scenarios. Similarly, 71% reported ease in determining reasonable next steps from their observations and diagnostic input, and 82% found making a preliminary diagnosis straightforward.

Recent reports suggest that immersive, VR-based medical training improves the users’ interaction with the learning material, leading to better knowledge acquisition [22, 23]. Therefore, we assessed how immersed students felt during the simulation. The majority found the simulation highly realistic; 77% affirmed its realistic setting, and 83% felt fully immersed. However, opinions on the realism of emergency situations varied: 41% felt it was like a real emergency, 28% did not, and 31% were neutral. Student perspectives on the interactions with the virtual patients also varied widely, with 41% finding it realistic while 27% did not. Overall, 84% of students found the VR simulation engaging enough to maintain focus during the seminar.

To evaluate students’ perceived learning outcomes in more detail, we incorporated a set of nine questions. 76% believed the VR simulation would improve their general response in real emergencies, but only 52% felt more confident handling such situations appropriately, while 18% did not. Additionally, 54% reported they could better prioritize in emergencies, while 15% did not feel better prepared for prioritization. Notably, 71% believed that VR practice would benefit their future medical careers. Overall, 91% viewed the VR simulation as an effective learning tool, and 88% considered it valuable for acquiring skills in the management of emergency situations.

Our final objective was to analyze student opinions on the overall structure of the seminar. Overall, the design was very well-received by the students: 94% of students found the seminar format appropriate. Another 94% appreciated the active case discussions in the seminar groups, led by student tutors. Taken together, 93% of the students expressed a desire for more such interactive teaching methods in medical education. Most importantly, our longitudinal subanalyses across all four cohorts demonstrated that these trends remained consistently stable over time (see Supplementary Fig. 1).

### Simulation sickness

**Fig. 3:**
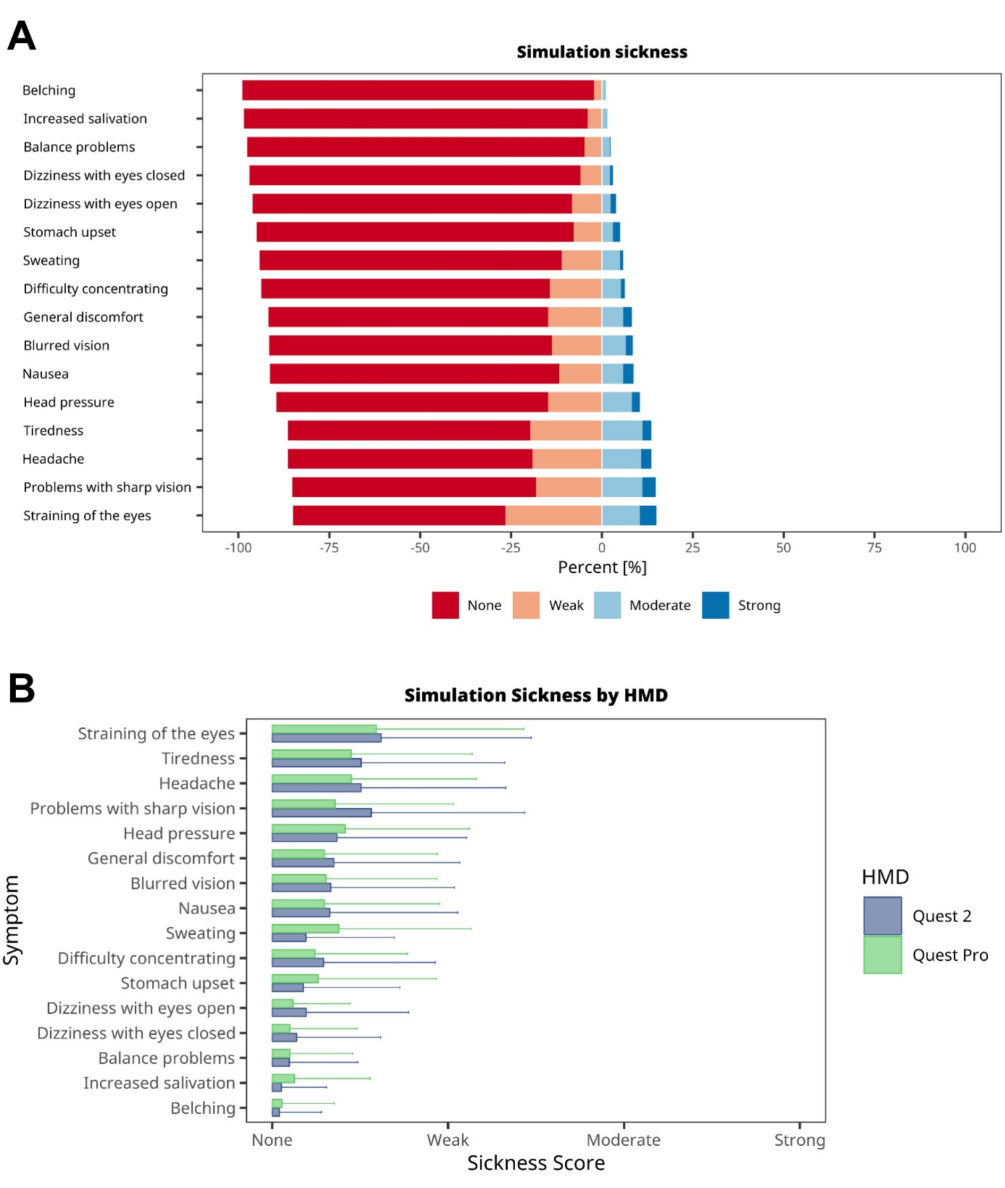
Evaluation of simulation sickness associated with VR device use. caused by wearing the VR devices **A**, Likert scale plots (4-point scale) showing the combined results for simulation sickness caused by wearing the VR devices. Bars represent the percentage of responses for each Liker score. **B**, Side-by-side comparison of simulation sickness symptoms associated with different head-mounted displays (HMDs). Data are presented as the mean ± standard deviation (SD) of the Likert score (4 points)

Previous reports have indicated instances of dizziness or cybersickness in users of VR HMDs, ranging from mild discomfort to severe symptoms, leading some participants to withdraw from studies [24–26]. However, advancements in XR/VR technology have significantly lowered the incidence of such issues [27]. In our own experience, Meta Quest 2 HMDs tend to be associated with a very low occurrence of simulation sickness symptoms. Nevertheless, the choice of software can also affect simulation sickness. Hence, we employed a well-established simulation sickness questionnaire [21] to assess symptoms related to using STEP-VR. Overall, the experienced symptoms were generally mild (Fig. 3A). Of 16 symptoms, 11 were reported as moderate or severe by fewer than 10% of students. More than 10% of students reported more than five symptoms (head pressure, headache, tiredness, vision sharpness issues, and eye strain) as moderate or severe. Vision sharpness and eye strain were most frequently cited as severe. Taken together, these findings indicate that both used types HMDs, in conjunction with STEP-VR, typically caused only mild to moderate simulation sickness symptoms.

We were also interested in whether newer HMD features, such as higher pixel density, increased field of view (FoV), larger inter-pupillary distance (IPD) range, and design improvements, may impact simulation sickness. Contrary to expectations, our findings showed no significant difference in simulation sickness intensity between the two HMD models that were used (Fig. 3B). However, the Meta Quest Pro showed a marginally lower incidence of sharp vision problems. In conclusion, our data indicate no significant disparity in simulation sickness occurrence between the Meta Quest 2 and Quest Pro HMDs.

### Ease of handling

**Fig. 4:**
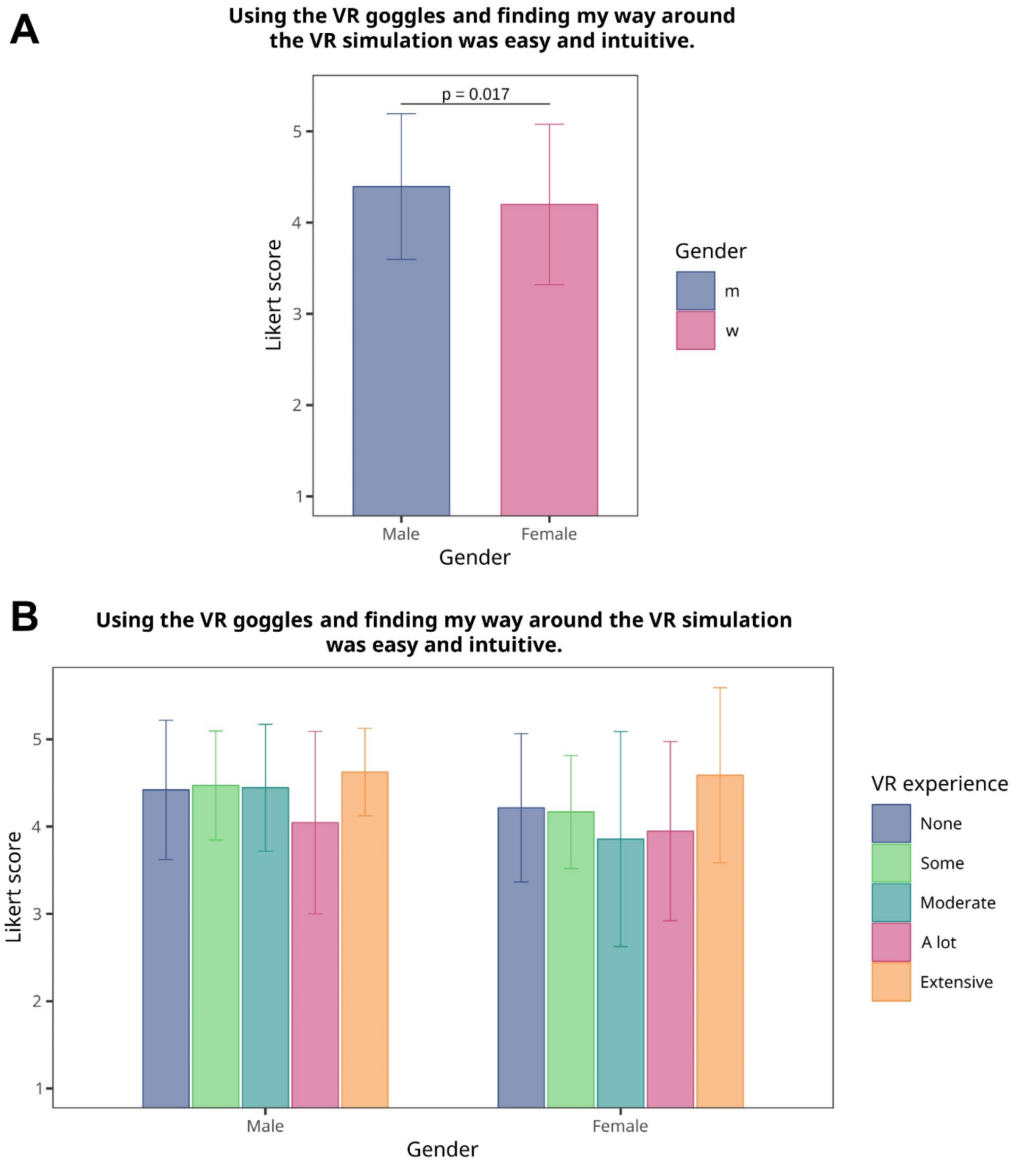
Self-reported ease of using VR-HMD and navigating the simulation. **A**, Comparison of Likert scores for the item *“Using the VR goggles and finding my way around the VR simulation was easy and intuitive.”* between male and female students. The Mann-Whitney *U* test was used to analyze differences between groups. Due to the low numbers of non-binary students ($\:n=3$), the analysis focused only on male and female students. **B**, Likert scores for above mentioned item, showing self-reported ease of use categorized by gender and previous VR experience. Bars represent the mean ± SD

Previous studies on VR in medical education have noted some reservations towards VR among female medical students, particularly those with a lower proclivity for computers [16, 28]. Hence, we were interested in evaluating whether female students would report a lower acceptance of the STEP-VR training tool, which could influence its curricular integration. We examined responses to the ease of using VR HMD and navigating the VR simulation, taking into account self-reported gender and prior VR experience. Our analysis revealed a statistically significant, yet exceedingly small, difference in ease of use between male and female students (*p* = 0.017, δ = 4.21 ⋅ 10^−5^, 95%*CI* 7.82 ⋅ 10^−5^ − 1.39 ⋅ 10^−5^) (Fig. 4A), suggesting that any concerns among female students regarding VR handling are minimal and likely do not represent a meaningful barrier. Further, when considering previous VR experience (Fig. 4B), ease of use scores were fairly consistent across genders, with a slight increase for both males and females with extensive VR experience. In summary, the differences in ease of handling between male and female students were minor and are likely inconsequential for incorporating VR-based emergency training into medical curricula.

## Discussion

Immersive technologies have only just started to meaningfully enrich our educational repertoire. Indeed, the potential impact of VR technology on practices in medical education has been highlighted in several recent studies [10–12, 15, 16, 29]. In a recent report for instance, small groups of medical students participated in highly immersive virtual clinical scenarios (STEP-VR) and were subsequently evaluated for acceptance, focusing on both psychological and educational aspects. The results suggested that active participants felt a strong sense of realism as well as didactic value, and the investigators concluded that curricular implementation of VR-based training sessions of medical emergencies may in fact be feasible [16]. However, this study faced limitations, including restriction to undergraduate students of only one cohort of one institution and, importantly, a tethered hardware setup that delimited liberty of action. Moreover, other studies have investigated VR-based teaching for pediatric emergencies and reported high levels of perceived usefulness and increasing levels of perceived competence among participants [29]. This study also had limitations, such as small sample sizes and a narrow focus on pediatric emergencies alone, limiting the generalizability of the reported findings.

Building on this foundation, the STEP-VR application was adopted and studied longitudinally across four consecutive student cohorts over a period of two years. This novel training concept, integrating XR/VR technology with a dynamic physiology system and discrete aspects of gamification, was tested for technical feasibility and tolerance of simulation sickness. Our study provides comprehensive data on key challenges related to the practical application of the learning format, students’ overall views on learning emergency medicine content with XR/VR assistance, and the occurrence of simulation sickness.

Most students reported a high level of engagement and a more profound understanding of complex medical conditions, attributing this to the immersive and interactive nature of VR. Importantly, the majority of students encountered no difficulties in operating the HMDs or the associated software.

Interestingly, while a majority of students experienced a strong sense of immersion, about one third did not feel the simulation closely mimicked a real emergency situation with a real patient. However, it was also shown that low-fidelity simulations – though less realistic – did not perform worse in learning compared to high-fidelity simulation [30]. Our study found that although the realism of the simulation was a recurring criticism, many students were still content with their overall learning experience. This indicates that the level of realism provided by the application may be sufficient for effective learning.

Recent studies have noted some reservation among female students towards VR, particularly for those with less prior VR experience [16, 28]. Concerning gender differences, female students in our study reported slightly lower ease of use with VR, but the difference was minor, implying potentially negligible impact on future curriculum integration.

Regarding cost-effectiveness in implementing XR/VR in medical education [25], we found no notable differences in average responses across various aspects between experiences with the high-end Meta Quest Pro versus the significantly less expensive Meta Quest 2 HMD model. Students using the Meta Quest Pro reported only slightly fewer issues with vision clarity. Overall, the less expensive Meta Quest 2 was deemed sufficient in our setting, balancing both cost and educational value.

Our study has notable limitations, hence any recommendation should come with a note of caution and underscore the necessity for additional research including prospective studies with long follow-ups. One limitation is that our study relied primarily on self-reported data and was performed at a single center, which limits the generalizability of the findings. Different student demographics or educational cultures in other institutions and countries might yield different results. Another limitation is the potentially misleading assumption that XR/VR-based training is universally suitable for all medical students. It’s important to explore how different students, with varying learning styles and technological aptitudes [31], interact with and benefit from these tools. There may be a subset of students who find XR/VR interfaces less intuitive or even disorienting; another concern is the potential introduction of cognitive load, all of which could impact their learning experience [25, 28].

Moreover, while the benefits of immersive technology in medical training may appear apparent, the practical challenges of implementation at any given teaching facility must be addressed. This includes the cost of equipment, the need for specialized technical support, ensuring that content aligns with learning objectives, and the well-known risk of rapid technological obsolescence of both hardware and software [25, 32]. Additionally, little is known about the long-term effects of repeated exposure to immersive environments including clinical settings. Finally, the reliance on self-reported data – as was the focus of our study – introduces the potential for response biases. Hence, it would be conducive to develop standardized and more objective metrics for assessing the effectiveness of XR/VR training, that ideally should include evaluating how well these “virtually acquired” skills transfer to real-world medical settings.

## Conclusions

In conclusion, our findings suggest that XR/VR-based simulation training for the management of patients with medical emergencies could benefit medical students in preparing them effectively for the complexities of emergency medical care. However, further research is needed to explore long-term outcomes of VR training on clinical practice and patient care. There is also a need to examine the scalability of such complementary programs and their integration into traditional medical curricula. If these aspects are addressed successfully, STEP-VR and similar XR/VR-based educational tools could indeed represent a paradigm shift in medical education, with implications extending far beyond emergency medicine.

**Table 1:** Demographic profile of survey participants

*Gender – no.*		
Male	171	33.4%
Female	338	66.0%
Non-binary	3	0.5%
***Age – years***		
21–25	352	71%
26–30	101	19%
> 30	44	9%
Mean (standard deviation)	25.2 (2.8)	-
Min < Median < Max	21 < 24 < 36	-
***Round – no.***		
Winter semester 2021/22	142	26.8%
Summer semester 2022	119	22.5%
Winter semester 2022/23	137	25.9%
Summer semester 2023	131	24.8%
***Head-mounted display***		
Meta Quest 2	398	75.2%
Meta Quest Pro	131	24.8%
***Previous experience with VR– no.***		
None	342	65.9%
Some	56	10.8%
Moderate	30	5.8%
A lot	48	9.2%
Extensive	43	8.3%

## Electronic supplementary material

Below is the link to the electronic supplementary material.

Supplementary Material 1

## Data Availability

Data is provided within the manuscript or supplementary information files.
