# Supplementary material for: Immersive medical training: a comprehensive longitudinal study of extended reality in emergency scenarios for large student groups
Source: BMC Med Educ. 2024 Sep 9;24:978. doi: 10.1186/s12909-024-05957-3 (PMC11385130; doi:10.1186/s12909-024-05957-3)
Supplement: Supplementary file 1 — Supplementary Material 1 [file 12909_2024_5957_MOESM1_ESM.docx]

Supplementary Material

Immersive Medical Training: A Comprehensive Longitudinal Study of Extended Reality in Emergency Scenarios for Large Student Groups

Supplementary Table 1 | Design of the student’s survey.

| **Category** | **Question type** | **Question text** | **Cronbach's *α*** |
| --- | --- | --- | --- |
| Handling of the VR device | Likert (5 point) | I could see everything sharply through the VR glasses.  Using the VR goggles and finding my way around the VR simulation was easy and intuitive. | 0.64 |
| Complexity of content and challenges | Likert (5 point) | I knew enough to handle the emergencies.  It was easy for me to make a (suspected) diagnosis.  It was easy for me to deduce further procedures from the findings. | 0.76 |
| Degree of immersion | Likert (5 point) | The VR simulation creates a realistic learning environment.  I was totally immersed in the VR simulation.  The VR simulation was so engaging that it was easy for me to pay attention in the seminar.  I felt the interaction with the patient was very realistic.  I felt like I was in a real emergency situation. | 0.84 |
| Subjective learning success | Likert (5 point) | The VR simulation helps me to react better in real emergency situations.  The VR simulation helps me understand the physiological relationships in emergency situations.  The VR simulation is a good tool to acquire skills in emergency situations.  The VR simulation motivates me to further study the contents of the seminar.  If there were more corresponding offers, I could well imagine the VR simulation for further cases.  I think the VR simulation is a good learning tool.  I now have more confidence to respond appropriately in emergency situations. | 0.91 |

Supplementary Table 1 | *(continued)*.

| Subjective learning success *(continued)* | Likert (5 point) | I can now prioritize better in emergency situations.  Practicing the emergency scenarios in the VR simulation will help me in my future profession as a physician. |  |
| --- | --- | --- | --- |
| Seminar design | Likert (5 point) | The use of the goggles was well explained to me.  The design as a seminar format is suitable or beneficial.  The active discussion of the cases in the group (moderated by tutor, lecturer) was additionally instructive and useful.  I wish there were more teaching offers of this kind in medical teaching. | 0.78 |
| Simulation Sickness Questionnaire | Likert (4 point) | *For every symptom, please state in which intensity it appears in this moment:*  General discomfort  Tiredness  Headache  Straining of the eyes  Problems with sharp vision  Increased salivation  Sweating  Nausea  Difficulty concentrating  Head pressure  Blurred vision  Dizziness with eyes open  Dizziness with eyes closed  Balance problems  Stomach upset  Belching | - |


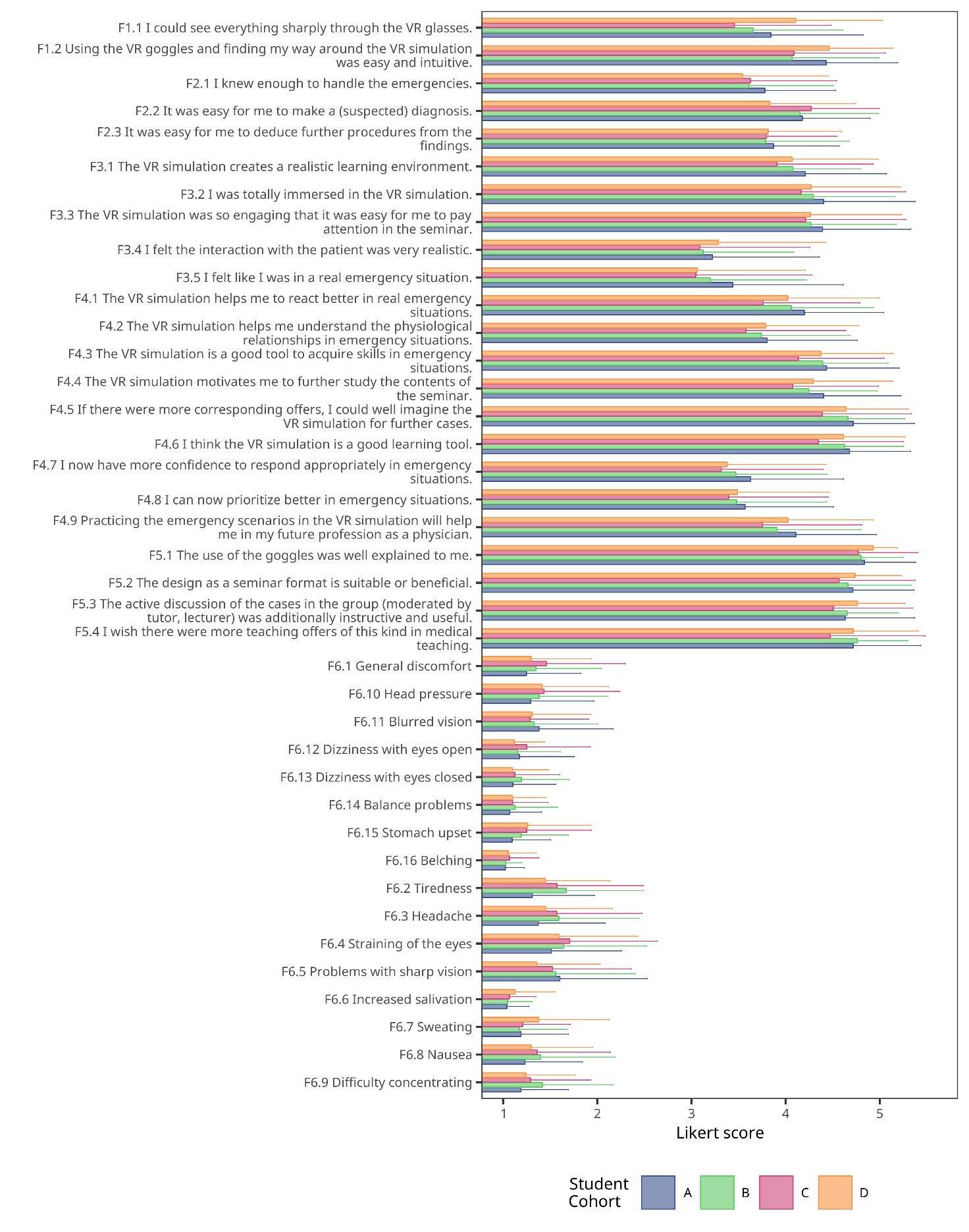


**Supplementary Figure 1 | Comparison of the responses by student cohort.** Mean ± standard deviation is shown.
